# Four-dimensional ultrasound versus torsional ultrasound phacoemulsification in cataracta nigra: a single-nucleus *ex vivo* bench pilot

**DOI:** 10.3389/fopht.2026.1795898

**Published:** 2026-04-16

**Authors:** Rosa Giglio, Daniele Tognetto

**Affiliations:** Department of Medicine, Surgery and Health Sciences, University of Trieste, Trieste, Italy

**Keywords:** cataracta nigra, cumulative dissipated energy, *ex vivo* bench study, four-dimensional ultrasound, phacoemulsification, torsional ultrasound

## Abstract

Cataracta nigra, an extremely dense nuclear cataract, often requires prolonged ultrasound use during phacoemulsification, increasing the risk of corneal endothelial injury and incision-site thermal damage. We compared the Alcon UNITY Cataract System using 4-dimensional (4D) ultrasound with the Alcon CENTURION Vision System with torsional ultrasound in an ex vivo cataracta nigra bench model. A single human cataracta nigra nucleus was divided into four fragments; two fragments were emulsified with UNITY and two with CENTURION under identical user-selected console settings (35% power; IOP 36 mmHg; vacuum 450 mmHg; aspiration flow 30 mL/min). Endpoints included emulsification time, effective phaco time (EPT), cumulative dissipated energy (CDE), and qualitative video assessment of fragments behavior. In this single-specimen model, compared with CENTURION, UNITY showed shorter emulsification time (mean 12.0 s vs 43.5 s) and lower console-reported energy indices (mean EPT 4.2 s vs 15.2 s; mean CDE 1.12 percent-seconds vs 15.01 percent-seconds). Video review suggested more continuous fragment engagement with minimal chatter using UNITY, whereas CENTURION more often showed initial fragment repulsion and turbulence. Because EPT and CDE are console-derived, these findings should be interpreted as relative performance under matched user-selected settings rather than equivalent physical energy delivery. These preliminary findings suggest that the 4D ultrasound mode might be associated with improved emulsification efficiency compared to torsional mode in ultra-dense nuclear material. Further studies using additional specimens and clinical cohorts are needed to determine whether these bench findings translate into meaningful surgical benefit.

## 1 Introduction

Cataracta nigra is an advanced nuclear cataract characterized by a very dense nucleus that appears dark brown to black. Phacoemulsification in these lenses often requires prolonged ultrasound exposure, increasing the risk of endothelial stress and incision-site thermal burns (1, 2). Manual extracapsular techniques avoid the use of ultrasound in very dense cataracts but may require a larger incision (3). Phacoemulsification efficiency is influenced by ultrasound stroke geometry, frequency and energy/stroke modulation. Longitudinal ultrasound generates axial forward-backward motion, which may increase nuclear repulsion, whereas torsional ultrasound produces lateral shearing motion that has been associated with reduced chatter and improved followability (4–6). Hybrid or multi-vector systems introduce additional stroke modulation intended to improve cutting efficiency and fluidic stability. In contrast to torsional ultrasound, which primarily produces lateral shearing oscillation around the longitudinal axis of the tip, 4D ultrasound combines axial and lateral components with dynamic stroke modulation to improve cutting efficiency while maintaining fragment followability, the tendency of nuclear pieces to remain engaged with the phaco tip. The Alcon UNITY Cataract System combines pressure-controlled fluidics with 4D ultrasound (7). Bench and ex vivo models have been widely used to evaluate cumulative dissipated energy (CDE) and thermal dynamics under controlled conditions (8–10). CDE and effective phaco time (EPT) are console-derived indices that vary by modality and platform generation (10, 11). This ex vivo pilot study compared UNITY (4D ultrasound) with CENTURION (torsional ultrasound) under standardized user-selected console settings. The aim was to evaluate whether 4D ultrasound could be associated with greater emulsification efficiency compared to torsional mode in an ultra-dense cataract model.

## 2 Methods

A single human cataracta nigra nucleus was obtained en bloc during a routine extracapsular cataract extraction (ECCE). Written informed consent for research use of discarded tissue was obtained in accordance with institutional ethical policies. After ECCE, the nucleus was divided with a single-use steel dissecting blade. The nucleus was first bisected into two hemi-nuclei, and each hemi-nucleus was then divided again to obtain four fragments. The four fragments were visually inspected and appeared to be approximately comparable in size and thickness, although no formal mass or volumetric measurements were obtained. To balance potential intranuclear heterogeneity, each phaco system was assigned one fragment from each hemi-nucleus (i.e., one fragment per half per system). Two fragments were emulsified with the Alcon UNITY Cataract System (4D ultrasound mode) and two with the Alcon CENTURION Vision System (torsional ultrasound mode). Both systems were equipped with the Active Sentry handpiece (Alcon, Fort Worth, TX, USA). The experiments were performed using a transparent test chamber filled with balanced salt solution (BSS). Between runs, fragments were kept fully submerged in fresh BSS at room temperature to limit dehydration. The phaco handpiece was fixed vertically in a custom holder to standardize tip position. Each fragment was aspirated to stable occlusion, and ultrasound energy was delivered only when the phaco tip was occluded by lens material. An ultrasound power setting of 35% was selected for both CENTURION (torsional mode) and UNITY (4D mode) as a stable moderate setting for dense nuclear emulsification. This setting allowed sustained tip engagement in the bench setup. Other settings included target intraocular pressure (IOP) 36 mmHg, vacuum limit 450 mmHg, and aspiration flow rate 30 mL/min. All runs were performed by the same operator (DT). The endpoint was complete emulsification and aspiration, defined as the absence of visible residual nuclear material.

For each experimental run, console-derived metrics were recorded: total emulsification time, EPT and CDE. EPT represents the equivalent ultrasound time at 100% power that would correspond to the same console-calculated energy exposure at the selected percentage amplitude. CDE was recorded as displayed by each console and expressed in percent-seconds. CDE is a manufacturer-defined, modality-specific index derived from console parameters rather than a direct physical measurement of ultrasonic energy in joules. Historically, the CDE algorithm was engineered as a surrogate for the thermal energy dissipated at the incision site, proportional to the physical displacement of the phaco tip barrel, to monitor the risk of thermal wound injury. For the CENTURION system in torsional mode, manufacturer documentation defines CDE as torsional amplitude (%) × torsional time (seconds) × 0.4, where 0.4 is a manufacturer-derived coefficient accounting for the relative cutting efficiency and stroke characteristics of torsional motion compared with longitudinal ultrasound. While the Unity 4D system utilizes a multi-vector oscillatory motion at the distal tip to enhance cutting efficiency, the mechanical displacement of the tip barrel at the wound site is supposed to remain consistent with standard torsional motion. To our knowledge, the precise mathematical formulation used by UNITY has not been publicly disclosed in manufacturer documentation or peer-reviewed literature. However, in UNITY (4D mode), CDE is similarly presented as a cumulative amplitude-weighted index expressed in percent-seconds. The comparability of CDE between the two machines was examined through a standardized testing in a BSS-filled chamber. Ultrasound was delivered in continuous torsional (Centurion) and 4D (Unity) modes at fixed power increments (25%, 50%, and 100%) for durations of 1.0 and 2.0 seconds (s) using an external timer. Measurements were retained if the activation time was within +0.2 s of the target duration. Thus, recordings from 1.0 up to 1.2 s were included in the 1-second group, and recordings from 2.0 up to 2.2 s were included in the 2-second group due to an expected potential manual latency between foot-pedal deactivation and the cessation of the console’s internal timer. These trials aimed to confirm whether both consoles report comparable CDE values for identical power and time parameters at the barrel level.

In our setting, the nuclear fragments were not prospectively weighed or measured volumetrically, therefore, normalization of time or energy metrics per unit mass was not performed. Videos were reviewed for fragment chatter and chamber turbulence. Given the single-specimen pilot design (n=1 nucleus), no formal statistical analysis was performed, and results are reported descriptively.

## 3 Results

For nuclear fragments emulsification tests, two runs were completed for each system under identical user-selected settings (**Table 1**). Mean emulsification time was lower with UNITY than with CENTURION (12.0 vs 43.5 s;approximately 72% lower). Mean EPT was likewise lower (4.20 vs 15.23 s, approximately 72% lower), as was mean CDE (1.12 vs 15.01 percent-seconds, approximately 92% lower). On qualitative video review, UNITY showed more stable fragment followability with minimal chatter, whereas CENTURION more frequently exhibited initial fragment repulsion and mild turbulence associated with occlusion breaks. Illustrative runs from both modalities are provided (**Supplementary Videos 1**, **2**). Results of the CDE comparability tests are summarized in **Table 2**.

**Table 1:** Performance metrics for each system and fragment.

System	Run (fragment)	Time (s)	Ultrasound power (%)	EPT (s)^a^	CDE (percent-seconds)^b^
UNITY (4D ultrasound mode)	Run 1 (Fragment 1)	11	35	3.85	1.03
UNITY (4D ultrasound mode)	Run 2 (Fragment 2)	13	35	4.55	1.21
CENTURION (torsional ultrasound mode)	Run 3 (Fragment 3)	42	35	14.70	14.49
CENTURION (torsional ultrasound mode)	Run 4 (Fragment 4)	45	35	15.75	15.53

^a^EPT is the effective phaco time (seconds), defined as the equivalent ultrasound time at 100% power corresponding to the console-calculated exposure at the stated amplitude.

^b^CDE is cumulative dissipated energy (percent-seconds), a console-derived cumulative energy index.

**Table 2:** Comparison of CDE values for UNITY 4D and CENTURION torsional modes at defined power and activation durations.

Duration	Ultrasound power (%)	CDE (percent-seconds)^a^	
		Unit (4D ultrasound mode)	Centurion (torsional ultrasound mode)
1.0 s^b^	25	0.07	0.09
	50	0.21	0.24
	100	0.43	0.46
2.0 s^b^	25	0.19	0.20
	50	0.30	0.44
	100	0.50	0.79

^a^ CDE is cumulative dissipated energy (percent-seconds), a console-derived cumulative energy index.

^b^ Activation times represent target durations with an allowed tolerance of up to +0.2 s only.

## 4 Discussion

In this single-nucleus ex vivo pilot bench model of an extremely dense cataract, the UNITY system in 4D ultrasound mode was associated with shorter emulsification times and lower console-reported energy indices than the CENTURION system in torsional mode under identical user-selected settings. We recorded a reduction in CDE (1.12 vs. 15.01 percent-seconds) and emulsification time (12.0 s vs. 43.5 s) with the UNITY 4D system, corresponding to a 92% reduction in CDE and a 72% reduction in emulsification time compared to the CENTURION system under the conditions tested. Internal manufacturer data regarding UNITY 4D reported a 48% reduction in CDE and a 41% reduction in total energy with a 2 times faster nucleus removal (12). Our bench results suggest that the efficiency gains associated with multi-vector oscillatory motion may be even more pronounced under standardized experimental conditions. The CDE comparability tests suggested that the energy delivered at the incision site per second, represented by the mechanical displacement of the tip barrel, was broadly comparable between standard torsional and 4D modes. For a 1.0 s burst at 100% power, both systems yielded CDE values (0.43 vs 0.46 percent-seconds for UNITY and CENTURION, respectively) that were close to the theoretical torsional constant of 0.40. A divergence in performance was observed at the 2.0-second interval at 100% power, where the UNITY 4D system recorded a CDE of 0.50 percent-seconds compared with 0.79 percent-seconds for the CENTURION. Although the reasons for this difference cannot be determined from the present pilot study, the lower CDE recorded by the UNITY 4D system may reflect integrated thermal-protective attenuation, dynamic stroke modulation, or another form of output regulation designed to limit continuous energy dissipation at the incision. Within the limits of this experimental model, the marked reduction in overall CDE observed in our sample of cataracta nigra fragments may therefore be related less to energy delivery per unit time at the incision and more to improved cutting efficiency at the distal tip. In this context, the 4D volumetric motion may have allowed more efficient fragment emulsification, thereby reducing the total ultrasound-on time required and, consequently, the cumulative energy delivered. Qualitative review of the recorded runs supports the hypothesis of improved efficiency, as UNITY maintained more continuous engagement with minimal chatter, whereas CENTURION more frequently exhibited initial fragment repulsion and transient turbulence at occlusion break. Historically, CDE was developed primarily as a surrogate marker of incisional thermal safety and the risk of wound burn (13). However, total intraocular energy, expressed in joules, may represent a more relevant indicator of endothelial cell stress and postoperative corneal edema than console-specific indices alone. While CDE reflects amplitude-weighted time at the wound, the actual physical work performed by the distal tip within the anterior chamber, together with associated chatter or fluidic turbulence, may be more directly related to endothelial cell loss (5, 14–16). This is particularly relevant in very hard nuclei, in which increased ultrasound use has been associated with endothelial stress and early corneal edema (2). This pilot study has several limitations, including its reliance on a single cataracta nigra nucleus that was manually divided. Although allocation was balanced so that each modality was tested on one fragment from each hemi-nucleus, residual fragment-level variability in geometry, thickness, and brittleness cannot be excluded. Furthermore, because fragments were not prospectively weighed or volumetrically measured, normalization of time or energy indices per unit lens mass was not feasible. This study standardized console settings rather than calibrating physical stroke amplitude or directly measuring delivered energy. The selected parameters (35% power, IOP 36 mmHg, vacuum 450 mmHg, aspiration flow 30 mL/min) were chosen to provide stable occlusion and sustained emulsification, thereby allowing assessment of modality behavior under matched user-selected conditions. A multi-parameter analysis across varying power and fluidic settings was beyond the scope of this single-specimen pilot and would require a separate protocol using independent specimens. Confirmation in additional nuclei or standardized dense surrogate materials will be required to assess reproducibility and enable mass-normalized comparisons, as in prior laboratory-based thermal and efficiency studies (8–10). As an ex vivo bench model, this setup does not replicate capsular bag dynamics, incision architecture, corneal thermal dissipation, or physiologic intraocular fluidics. Because fluidic parameters are known to influence intraoperative dynamics and postoperative outcomes (17, 18), further study under varied conditions is warranted. Future experimental studies will be needed to move beyond manufacturer-specific indices and focus on absolute total energy in joules and measures of volumetric energy distribution.

In summary, within the constraints of a single-nucleus pilot observation under standardized console inputs, UNITY 4D ultrasound was associated with shorter emulsification time, lower console-derived energy indices, and less fragment chatter. These findings are consistent with the hypothesis of improved mechanical efficiency in ultra-dense nuclear material; however, validation in additional specimens and clinical studies is required before any inference regarding endothelial stress or surgical safety can be made.

## Data Availability

The raw data supporting the conclusions of this article will be made available by the authors, without undue reservation.

## Supplementary material

Supplementary Video 1 UNITY 4D mode Run 2 (Fragment 2) in the bench test chamber, showing continuous fragment engagement with no obvious visible chatter during emulsification of the cataracta nigra fragment.

Supplementary Video 2 CENTURION torsional mode Run 4 (Fragment 4) in the bench test chamber, showing apparent fragment repulsion and chamber turbulence at ultrasound initiation and occlusion breaks.
